# Neural Substrates of Incidental Associations and Mediated Learning: The Role of Cannabinoid Receptors

**DOI:** 10.3389/fnbeh.2021.722796

**Published:** 2021-08-06

**Authors:** Christina Ioannidou, Arnau Busquets-Garcia, Guillaume Ferreira, Giovanni Marsicano

**Affiliations:** ^1^INSERM, U1215 Neurocentre Magendie, Bordeaux, France; ^2^University of Bordeaux, Bordeaux, France; ^3^Integrative Pharmacology and Systems Neuroscience Research Group, Neurosciences Research Program, IMIM (Hospital del Mar Medical Research Institute), Barcelona, Spain; ^4^INRAE, Nutrition and Integrative Neurobiology, Bordeaux, France

**Keywords:** CB1, endocannabinoids, higher-order conditioning, sensory preconditioning, incidental learning, incidental associations, mediated learning

## Abstract

The ability to form associations between different stimuli in the environment to guide adaptive behavior is a central element of learning processes, from perceptual learning in humans to Pavlovian conditioning in animals. Like so, classical conditioning paradigms that test direct associations between low salience sensory stimuli and high salience motivational reinforcers are extremely informative. However, a large part of everyday learning cannot be solely explained by direct conditioning mechanisms – this includes to a great extent associations between individual sensory stimuli, carrying low or null immediate motivational value. This type of associative learning is often described as incidental learning and can be captured in animal models through sensory preconditioning procedures. Here we summarize the evolution of research on incidental and mediated learning, overview the brain systems involved and describe evidence for the role of cannabinoid receptors in such higher-order learning tasks. This evidence favors a number of contemporary hypotheses concerning the participation of the endocannabinoid system in psychosis and psychotic experiences and provides a conceptual framework for understanding how the use of cannabinoid drugs can lead to altered perceptive states.

## Introduction

In order to make decisions in daily life, we often rely on our previous experiences. We tend to repeat actions that were profitable in the past and, conversely, to avoid those that led to negative consequences. Therefore, the vast majority of learning and memory studies tends to focus on similar situations, where a neutral stimulus (i.e., carrying low salience levels *per se*) is directly associated with a biologically significant, highly salient stimulus (food, electric shock, etc.), producing a new learned response in the individual. However, more often than not, in our environment we are exposed to novel and ambiguous settings, where direct experience is sparse and where a more flexible approach is required to predict – or guess – how a decision might turn out. In reality, while we engage in a particular activity, we are simultaneously surrounded by many incidental associations that might as crucially influence our future choices, as our direct experiences. Both humans and other animals have been shown to learn about the external world using such associations, often with the involvement of similar neural systems. Contrary to classical conditioning that generally produces solid and long-lasting responses, the memory of incidental associations is intrinsically labile. Rather than providing the individuals with direct information about the external world, it involves a large degree of “ambiguity.” Such ambiguity provides a level of flexibility that may be highly adaptive in changing environments. However, forming a mental association between two stimuli could also prove less beneficial if their co-occurrence is simply by chance and doesn’t represent truly an association. Incidental information is therefore constantly weighed against expectancies and environmental input to test its adherence to reality. Thus, whereas the ability to form incidental associations offers a way to better respond to unpredictable future challenges, a failure to precisely revise them and update them according to incoming information can account for “learning errors.” This can be observed for example in individuals who experience psychotic symptoms like delusions, who can rapidly accept incidental stimuli and events as meaningful and link them in unusual ways.

Understanding the cognitive and neurobiological mechanisms underlying these processes can therefore provide valuable insight both into the complicated abstract ways we learn, as well as into a potential source of cognitive dysfunctions in many mental illnesses. On that account, non-human animal models of incidental learning are crucial in contemporary neuroscientific research.

## Higher-Order Conditioning, Incidental Associations and Representation-Mediated Learning

Classical Pavlovian conditioning described how the brain represents dependent relationships between environmental stimuli and still remains the best-characterized associative learning model (Pavlov, 2010). In first-order Pavlovian conditioning, a conditioned stimulus (CS, such as a tone or light) acquires motivational significance by being paired with an intrinsically rewarding or aversive unconditioned stimulus (US, such as food or foot shock). Learning is evaluated by the ability of the CS to elicit a conditioned response (CR) in anticipation of the occurrence of the US. Although traditional views for Pavlovian conditioning described it as the transfer of an unconditioned reflex from the US to the CS, most contemporary learning theories agree that it involves the establishment of associations between internal memory representations of the CS, US, and their relationship (Fanselow and Wassum, 2015).

Although extremely informative, Pavlovian first-order conditioning is not sufficient for representing more ambiguous situations, such as the majority of the ones occurring in every-day life. In fact, a large part of the learning processes to represent our external world involves higher-order conditioning based on associations between low salience sensory stimuli, whose simultaneous or contiguous occurrence is stored because of its potential value for future choices. In higher-order conditioning a CS (S2) acquires associative strength by being paired with another CS (S1), rather than with a US. Two higher-order conditioning paradigms have been mainly used to assess higher-order conditioning in humans and animals, second-order conditioning, and sensory preconditioning. In second-order conditioning, the S1–S2 pairing can occur after S1 has been paired with the US, whereas in sensory preconditioning S1–S2 pairing precedes the S1-US (Gewirtz and Davis, 2000). In both cases subjects eventually display a conditioned response to a stimulus that was never explicitly paired with the reinforcer and thus higher-order conditioning tasks have been largely used to evaluate forms of indirect learning.

Sensory preconditioning in particular represents the most common behavioral protocol for studying incidental associations among relatively neutral or low-salience stimuli. In a typical experiment, two low-salience stimuli are first presented jointly during a preconditioning phase (S1–S2), then followed by classical conditioning of one of these stimuli by pairing it with a biologically meaningful (high salience) unconditioned reinforcer, like food or a foot shock (S1-US). Finally, exposing the subjects to either of the original stimuli (the one directly associated with the reinforcer and the one never associated) reveals the retrieval of direct and indirect memories, respectively (Brogden, 1939). Across a range of species (Karn, 1947; Hall and Suboski, 1995; Kojima et al., 1998; Muller et al., 2000; Wimmer and Shohamy, 2012), subjects’ response to the indirect preconditioned stimulus (S2) is found to be similar to that evoked by the directly conditioned cue (S1), assuming an association between the two has been formed.

Two prominent theoretical accounts are generally applied to explain the cognitive processes that underlie sensory preconditioning: the first one is the “associative chain” model, where the different associations are formed during the first and second phases of training allowing inference at test. In this account, the S1–S2 learning (phase 1) and the S1-US learning (phase 2) occur independently of each other and memories are integrated at the time of the testing, by recalling the two associations in order to infer on-the-fly the outcome that will likely follow (Rizley and Rescorla, 1972; Jones et al., 2012; Sharpe et al., 2017a; Sadacca et al., 2018; Wong et al., 2019; Wang et al., 2020). The second account, does not require memory integration at the time of testing, and refers to a process through which the preconditioned stimuli directly acquire positive or negative value during conditioning, due to a “unified representation” of S1 and S2. Through this process, often termed mediated or representation-mediated learning, presentation of S2 during the second phase of training activates a mental representation of S1, so that that this associatively retrieved memory might become further associated with the experience of the US. Eventually presentation of the S1 during test, retrieves this mediated S1-US association, and thus, elicits the observed response (Holland, 1981b; Hall, 1996; Wheeler et al., 2008; Wimmer and Shohamy, 2012; Schlichting and Preston, 2015; Lin and Honey, 2016). Representation-mediated learning was originally described by Holland (1981a, 1990), whose work demonstrated that animals can learn not only about directly perceived stimuli, but also about indirect, associatively retrieved representations of that stimuli. Auditory or visual stimuli (Holland, 1981a) or contexts (Dwyer, 1999, 2001) were initially paired with a flavored solution. When the tone, light or context were later paired with a gastric malaise, they served as substitutes for their associated flavor stimuli. This paradigm differs from a classical sensory preconditioning task in that these stimuli (tone, light, or context) did not form any appreciable first-order association with the illness, however, the associatively activated taste representations did support taste-aversion learning.

## Brain Regions Involved in Incidental Learning

Imaging studies in humans as well as experiments in rodents have provided insights into a network of brain regions that are involved in sensory preconditioning. The orbitofrontal cortex (OFC) has been shown to be necessary for forming value-neutral sensory associations, since both entire and selective inactivation of the OFC impairs inference about previously acquired stimulus-stimulus associations during the testing phase of sensory preconditioning (Jones et al., 2012). Moreover, single-unit recording experiments showed that neural activity in the lateral OFC reflects the acquisition of the associative information during the initial phase of training (Sadacca et al., 2018), and that optogenetic silencing of the OFC during this phase completely eliminates responding to the preconditioned cue during testing (Hart et al., 2020). Other structures, like the perirhinal and retrosplenial cortices have also been implicated. Lesions of the perirhinal cortex or its inactivation during preconditioning abolished sensory preconditioning (Nicholson and Freeman, 2000; Holmes et al., 2013; Wong et al., 2019), whereas chemogenetic silencing of the retrosplenial cortex during the preconditioning phase prevented inference at test without influencing direct conditioning (Robinson et al., 2014).

Interestingly, all aforementioned cortical regions are directly and indirectly interacting with the hippocampus (Agster and Burwell, 2013; Ritchey et al., 2015; Witter et al., 2017). Decades of research have characterized how the hippocampus critically contributes to representing and processing both real and abstract associative information (Port et al., 1987; Manns and Eichenbaum, 2009; Zeithamova et al., 2012; Voss et al., 2017) and many studies have highlighted its importance in sensory preconditioning both in humans (Bornstein and Daw, 2012, 2013; Wimmer and Shohamy, 2012; Shohamy and Turk-Browne, 2013) and in animals (Iordanova et al., 2009, 2011; Wheeler et al., 2013; Lin et al., 2016; Barron et al., 2020). Recent work additionally shows that a crosstalk between the hippocampus and the orbitofrontal cortex is important for inferring future outcomes during sensory preconditioning (Wang et al., 2020). Notably, in some studies, hippocampal activation has been demonstrated during the testing phase, suggesting its involvement primarily in the retrieval of the sensory-sensory associations (Talk et al., 2002; Barron et al., 2020). However, in other studies, hippocampal activation has been also shown during the conditioning phase of sensory preconditioning, as well as during the initial stimulus-stimulus associations, supporting a widespread hippocampal involvement and suggesting that this brain region may be particularly important not only for retrieval but also for the encoding of the incidental associations between neutral stimuli (Wang et al., 2020). This is consistent with evidence showing that the hippocampus is essentially involved in the acquisition of information, which can then be used by different brain regions to guide flexible behavior (Elliott Wimmer and Büchel, 2019; Schuck and Niv, 2019). In the following paragraphs we argue that one possible mechanism for the formation of low-salience stimulus-stimulus associations in the hippocampus during sensory preconditioning is involving the tight regulation of hippocampal GABAergic interneurons by cannabinoid receptors.

## Cannabinergic Control of Incidental Associations

Originally discovered as the endogenous targets of the cannabis plant psychotropic derivative Δ^9^-tetrahydrocannabinol (THC), cannabinoid receptors and specifically type 1 cannabinoid receptors (CB1Rs) are key neuromodulatory elements of synapses. Physiologically, cannabinoid receptors are the main targets of endogenous signaling molecules called endocannabinoids, forming, together with the enzymatic machinery for their synthesis and degradation, the so-called endocannabinoid system (ECS) (Piomelli, 2003; Lu and Mackie, 2016). CB1 receptors are likely the most abundant G protein-coupled receptors in the brain, with amounts of protein comparable to NMDA and GABAA receptors (Herkenham et al., 1990; Howlett, 2002; Freund et al., 2003). The expression levels of CB1 receptors can drastically differ among different cell types and can diverge between different brain regions (Han et al., 2012; Busquets-Garcia et al., 2018a). In cortical areas such as the hippocampus and neocortex, both glutamatergic principal neurons and GABAergic interneurons contain CB1 receptors, with the latter expressing the highest levels (Marsicano and Lutz, 1999; Marsicano and Kuner, 2008). The ECS has been involved in many forms of direct learning such as fear conditioning through CB1R in the amygdala (Marsicano et al., 2002; Metna-Laurent et al., 2012), conditioned taste aversion through CB1R in insular cortex (Kobilo et al., 2007), conditioned odor aversion through CB1R in medial habenula (Soria-Gomez et al., 2015) or conditioned odor preference through CB1R in the anterior piriform cortex (Terral et al., 2019), among others. Interestingly, the involvement of the ECS in direct conditioning appears to be more prominent in the modulation of the behavioral expression of the acquired memory, rather than its formation (Kobilo et al., 2007). However, despite the fact that CB1 receptor plays crucial roles in different phases of learning and memory processes (Rueda-Orozco et al., 2008; Marsicano and Lafenêtre, 2009; Akirav, 2011; Drumond et al., 2017), not many studies have addressed the physiological role of endocannabinoid signaling in higher-order learning.

In our previous work (Busquets-Garcia et al., 2018b) we evaluated the role of CB1R during the formation of incidental associations, using two different sensory preconditioning protocols in mice. Mice were first preconditioned by repeated exposure to pairs of low- salience sensory stimuli (pairing of an odor with a taste, or a light with a tone) forming an association between them. On subsequent days, mice were classically conditioned to associate one of these sensory stimuli (but not the other) with either an aversive or an appetitive stimulus. At the time of testing, both the directly conditioned stimulus but also the incidental preconditioned stimulus produced an aversion/preference, indicating the acquisition of both direct learning and mediated learning, respectively. Using this task, we showed that CB1R blockade upon preconditioning impaired the expression of mediated learning, however, CB1R blockade (or activation) at the stage of the testing did not affect the response to the preconditioned cue, strongly arguing for a specific role of endocannabinoid signaling in the initial processing of incidental stimulus-stimulus associations. Importantly, this effect did not appear to be limited to the specific sensory modality of the stimuli – whether those were olfactory and gustatory, or visual and auditory. The involvement of the ECS in different experimental conditions suggests broad common mechanisms underlying higher-order learning processes independently of the sensory modalities used and of the nature (aversive or appetitive) of the reinforcer.

With the hippocampus being a key brain region for sensory preconditioning, we addressed the role of hippocampal CB1R in these processes. In mice lacking CB1Rs selectively in the hippocampus or in forebrain GABAergic interneurons, mediated learning was compromised, yet direct learning was unaffected. Further experiments revealed that CB1Rs in hippocampal GABAergic neurons are indeed crucial for incidental learning, demonstrating a physiological link between hippocampal GABAergic signaling and associative memory between low-salience events. In fact, the paired presentations of the low-salience sensory cues during the initial, preconditioning phase induced a specific protein synthesis-dependent enhancement of hippocampal CB1R expression and facilitated long-term synaptic plasticity at hippocampal inhibitory synapses, suggesting that incidental learning might involve synthesis of new CB1Rs in hippocampal interneurons (Busquets-Garcia et al., 2018b). Interestingly, midbrain dopaminergic signaling has been shown to be both necessary and sufficient for the formation of incidental associations (Sharpe et al., 2017b). Dopamine function is also tightly regulated by and regulating the hippocampus (Lisman and Grace, 2005), and recently CB1 receptors have been identified in a subpopulation of hippocampal D1R-positive interneurons, where they control memory processes (Oliveira da Cruz et al., 2020). Therefore it is possible that endocannabinoids modulate incidental learning at hippocampal level through dopaminergic circuits, and further research should address this hypothesis.

## From Incidental Learning to Reality Testing: A Role for CB1 Receptor Signaling

Contrary to classical conditioning between a conditioned stimulus and an unconditioned stimulus that generally produces solid and long-lasting responses, an elemental characteristic of incidental associations between stimuli is that they are intrinsically weak (McDannald and Schoenbaum, 2009). Several studies have shown that, when studied through sensory preconditioning paradigms, the establishment of incidental learning requires a certain amount of training/paired presentations between the preconditioned stimuli. Paradoxically though, extending this training or pairings during preconditioning abolishes its expression (Holland, 2005; Holland et al., 2008; Busquets-Garcia et al., 2017), suggesting that the sensitivity to incidental learning can change as training proceeds. One explanation for this phenomenon suggests that, with moderate preconditioning, animals form a unified mental representation of the different preconditioned stimuli (S1+S2). However, with prolonged exposure to the stimuli, the subjects acquire more information about these stimuli, allowing them to separate their specific sensory features and consequently their associated outcomes (McDannald and Schoenbaum, 2009). As the preconditioned cues are indeed separated entities in reality, researchers defined this process as “reality testing,” following the basis of reality monitoring, the ability of individuals to distinguish real from illusory patterns and associations (Johnson and Raye, 1981; McDannald and Schoenbaum, 2009). An important aim down the road is therefore to unravel the complex biological processes that allow animals to switch from a unified representation of the different stimuli to their discrimination as independent entities (“reality testing”).

Type 1 cannabinoid receptors appear to be a key element of this switch. Our studies using reality testing protocols revealed that cannabinoids could disrupt this fundamental adaptive process, since acute administration of the main psychoactive component of cannabis, THC, was shown to impair reality testing, through activation of hippocampal CB1Rs (Busquets-Garcia et al., 2017). Thus, there is a dual impact of hippocampal CB1R signaling: whereas a *minimal activation* of CB1Rs is required for incidental learning in order to form unified stimuli representations, their *excessive stimulation* impedes testing of the real nature of these representations (reality testing). The data collected so far indicate that there seems to be a descending gradient of CB1R signaling during the switch between incidental learning and reality testing. On one hand, ECS activity has to be sustained at the moment of forming incidental learning, during which individuals collect possible useful information from seemingly unrelated stimuli. On the other hand, CB1R signaling has to be reduced when this potential information is contrasted to reality. In other words, more ECS activity leads to the generation of “open possibilities,” whereas the “closing” of these possibilities when they do not adhere to reality requires a decrease of CB1R signaling.

## Concluding Remarks: From Imagination to Psychosis?

The formation of incidental associations can underlie particular human abilities such as imagination and creativity, which are characterized by the ability to assume connections between unrelated phenomena in order to construct new ideas and imagine future scenarios (Schacter et al., 2012; Uddin, 2021). Cannabis use and creativity are also often portrayed as linked (LaFrance and Cuttler, 2017), with their connection culturally and commonly accepted. Cannabis intoxication has been shown to promote divergent thinking, the ability to see connections between distant concepts and reveal something new (Eisenman et al., 1980; Morgan et al., 2010), but at the same time to impair convergent thinking, the ability to reason based on logical inference (Oomen et al., 2018). This disparity could result in connections being made between seemingly unrelated concepts or ideas, which are then linked together and elaborated upon, a characteristic of creative thinking but also of the development of a delusional system, often present in psychiatric conditions such as schizophrenia and psychosis. Interestingly, the reconceptualization of schizophrenia symptoms as aberrant perceptions (hallucinations) (Corlett et al., 2019) and beliefs (delusions) (Feeney et al., 2017), has provided the framework to be studied through associative learning tasks in both humans and animals (Powers et al., 2017; Dwyer, 2018; Koh and Gallagher, 2020). Indeed, impaired “reality testing” was recently demonstrated in several animal models of schizophrenia in a way that mimics psychotic-like percepts (McDannald et al., 2011; Kim and Koh, 2016; Busquets-Garcia et al., 2017; Koh et al., 2018; Fry et al., 2019), with recent evidence suggesting that such phenomena involve dopamine signaling (Schmack et al., 2021).

Cannabis has been linked to the development of psychotic symptoms since a long time (Zuardi, 2006) and is well known to produce a range of immediate-onset psychotomimetic symptoms (Solymosi and Kofalvi, 2017), while alterations in the endocannabinoid system have also been implicated to the pathogenesis of schizophrenia and similar psychotic disorders (Muller-Vahl and Emrich, 2008). Given the general importance of the endocannabinoid system in the modulation of sensory perception (Soria-Gomez et al., 2014) and the fact that this function is centrally altered in psychotic states, it has been suggested that one important mechanism of cannabinoid-induced psychoses is linked to the alteration of perception of the external world. We therefore argue that the control of cannabinoid receptors over the formation and updating of incidental associations is contributing in orchestrating learning and associative thinking, in a continuum from normal perception to altered perceptual states.

## Author Contributions

All authors listed have made a substantial, direct and intellectual contribution to the work, and approved it for publication.

## Conflict of Interest

The authors declare that the research was conducted in the absence of any commercial or financial relationships that could be construed as a potential conflict of interest.

## Publisher’s Note

All claims expressed in this article are solely those of the authors and do not necessarily represent those of their affiliated organizations, or those of the publisher, the editors and the reviewers. Any product that may be evaluated in this article, or claim that may be made by its manufacturer, is not guaranteed or endorsed by the publisher.
